# Stearoyl-CoA Desaturase-1 Enzyme Inhibition by Grape Skin Extracts Affects Membrane Fluidity in Human Colon Cancer Cell Lines

**DOI:** 10.3390/nu12030693

**Published:** 2020-03-04

**Authors:** Valeria Tutino, Isabella Gigante, Maria Principia Scavo, Maria Grazia Refolo, Valentina De Nunzio, Rosa Anna Milella, Maria Gabriella Caruso, Maria Notarnicola

**Affiliations:** 1 Laboratory of Nutritional Biochemistry, National Institute of Gastroenterology “S. de Bellis” Research Hospital, 70013 Castellana Grotte, Italy; valeria.tutino@irccsdebellis.it (V.T.); isabella.gigante87@gmail.com (I.G.); valentinadx@hotmail.it (V.D.N.); 2 Personalized Medicine Laboratory, National Institute of Gastroenterology “S. de Bellis” Research Hospital, 70013 Castellana Grotte, Italy; maria.scavo@irccsdebellis.it; 3 Laboratory of Cellular and Molecular Biology, National Institute of Gastroenterology, “S. de Bellis” Research Hospital, 70013 Castellana Grotte, Italy; maria.refolo@irccsdebellis.it; 4 Research Centre for Viticulture and Enology, Council for Agricultural Research and Economics, 70010 Turi, Bari, Italy; rosaanna.milella@crea.gov.it; 5 Ambulatory of Clinical Nutrition, National Institute of Gastroenterology “S. de Bellis” Research Hospital, 70013 Castellana Grotte, Italy; gabriella.caruso@irccsdebellis.it

**Keywords:** Stearoyl-CoA desaturase-1, grape skin extracts, colon cancer cell lines

## Abstract

The polyphenolic compounds present in grape extracts have chemopreventive and anticancer properties. Here, we studied the ability of two grape skin extracts (GSEs), Autumn Royal and Egnatia, to influence the cell motility and membrane fluidity regulated by the enzyme Stearoyl-CoA desaturase-1 (SCD1) which increases with the cancer aggressiveness. Caco2 and SW480 human colon cancer cell lines were treated with increasing concentrations of GSEs to evaluate cell proliferation and motility. SCD1 levels were evaluated in both treated cell lines, by membrane lipidomic analysis conducted by gas chromatography. The expression levels of SCD1 and other factors involved in the reorganization of the cytoskeleton and focal adhesions were assessed by Real-time PCR, Western Blotting, and Immunofluorescence staining. High-performance liquid chromatography (HPLC) analyses were performed to determine the phenolic composition in the GSEs, finding them more expressed in Autumn Royal than in Egnatia. Both treatments reduced the levels of SCD1, phospho-Rac1/Cdc42/Rac1/Cdc42 ratio, Cofilin, Vimentin, and phospho-Paxillin especially in Caco2 compared to SW480, showing a different behavior of the two cell lines to these natural compounds. Our findings show that GSEs block the cell migration and membrane fluidity through a new mechanism of action involving structural cellular components.

## 1. Introduction

Metastasis is an active process in which cancer cells are able to move through the extracellular matrix (ECM) barriers thanks to local proteolysis, physical movement, lamellipodia, and filopodia formation. Some motility factors can contribute to the progression of metastases and to the increase of invasiveness by inducing rearrangements of the cytoskeleton, adaptations in cell adhesion, and stimulating epithelial to mesenchymal transition (ETM) [1,2,3].

Therefore, conformational cellular changes can be influenced both by a series of complex signaling pathways and by changes in the lipid profile of the plasma membrane. Lipids are a group of compounds that also influence different cellular functions, including cell membrane fluidity and cell morphology [4,5,6]. Several studies have shown that in tumor cells there is a greater demand for lipid biosynthesis to satisfy the growth and intensive proliferation of cells [7,8,9,10].

Stearoyl-CoA desaturase-1 (SCD1) is a key lipogenic enzyme that converts saturated fatty acids (SFAs), such as palmitic acid (C16:0) and stearic acid (C18:0), into monounsaturated fatty acids (MUFAs), such as palmitoleic acid (16:1n-7) and oleic acid (C18:1n-9). SCD1 activity can be estimated from the desaturation indices given by the palmitoleic acid/palmitic acid and oleic acid/stearic acid ratios [11,12]. The lipids derived from the unsaturation carried out by SCD1, are used to modulate membrane structure and fluidity; therefore, an important role of SCD1 in cell proliferation has been hypothesized [6,13]. Elevated levels of SCD1 and consequently of MUFAs in the lipid bilayer membranes have been detected in several tumors, such as colorectal cancer (CRC), and high levels of SCD1 have been positively related to the aggressiveness and malignancy of the disease [6,14,15]. Several SCD1 inhibitors have been proposed as possible anticancer agents; these molecules are able to reduce tumor formation, proliferation, migration invasion, and metastasis. Furthermore, there are molecules that induce apoptosis in different types of cancer by acting specifically on SCD1 [14]. The inhibition of SCD1 through natural compounds can result in health benefits [16,17,18]. The sterculic oil is an extremely precious natural product, capable of inhibiting SCD1 enzyme in obese ob/ob mice, reducing fat accumulation in the liver and adipose tissue and improving inflammation and insulin resistance, factors that create an environment conducive to the development of cancer [17].

Previously we have shown that diets enriched in olive oil and omega-3 polyunsaturated fatty acids (PUFAs) were able to modify the intestinal fatty acids profile in mice [19]. Higher levels of oleic acid and consequent reduction in Saturation Index levels (expressed as stearic acid/oleic acid ratio) were found in Apc^Min/+^ mice treated with olive oil compared to the control group fed standard diet, demonstrating that nutritional intervention can determine changes in membrane lipid content by acting on the activity of enzymes involved in lipid metabolism [19]. A reduction in stearic acid/oleic acid ratio levels in red blood cell membranes has been observed in different types of tumors and it seems to be associated with tumor mass and degree of malignancy [20,21,22].

In this context, numerous studies have been conducted in colon cancer cells to investigate the effects of grape extracts on the modulation of cell membrane components and on the viability and metastatic potential [23,24,25]. Grape skin and grape seed extracts are made up of different polyphenolic compounds that elicit multiple beneficial biological activities [26]. The resveratrol is able to accumulate sphingolipids in cell membranes, leading to cell cycle arrest and apoptosis [27]. Furthermore, in the Caco2 cell line, resveratrol reduces the global content of unsaturated fatty acids, probably acting on SCD1 [28]. Moreover, GSEs are able to down-regulate lipogenesis-mediating genes, such as SCD1 [29].

In light of these findings, in this work we wanted to evaluate the anti-metastatic effects of the grape skin extracts (GSEs) of two varieties of table grapes, Autumn Royal and Egnatia, on the lipidomic profile of the membranes of two colon cancer cell lines, Caco2 and SW480 with different degrees of differentiation. In particular, we wanted to demonstrate, for the first time, the ability of GSEs to modulate the membrane lipidomic profile by acting on SCD1. Moreover, we also wanted to study a possible role of Autumn Royal and Egnatia on cellular motility by evaluating different proteins involved in conformational cellular changes.

## 2. Materials and Methods

### 2.1. Grape Variety and GSEs Preparation

The grapes used in this study were the Autumn Royal, a seedless black grape variety, and the new Egnatia red seedless variety, planted and grown in Puglia at the Research Center for Viticulture and Enology of the Council for Agricultural Research and Economy, CREA-VE (Turi, BA, Italy). In order to ensure accurate sampling, grape samples were collected randomly and at maturity. The extraction of the phenolic fraction was carried out by treating the dried peel samples (250 mg of dry weight) with 5 mL of a solution of ethanol:water:hydrogen chloride 37% (70:30:1 *v/v/v*). Subsequently, after 24 h of complete darkness, the mixture was centrifuged, and the supernatant recovered and immediately analyzed.

### 2.2. Determination of Total Phenolic Content (TPC)

Autumn Royal and Egnatia varieties were chosen because they are rich in polyphenols and the determination of the TPC in GSEs was analyzed by the modified Folin-Ciocalteu colorimetric method as previously described [30]. The TPC included delphinidin-3-O-glucoside (Df3G), cyanidin-3-O-glucoside (Cy3G), petunidin-3-O-glucoside (Pt3G), peonidin-3-O-glucoside (Pn3G), malvidin-3-O-glucoside (Mv3G), malvidin-3-O-coumaroyl-glucoside (Mv3-coumaroyl-G), malvidin-3-O-glucoside equivalent 1 (Mv3G EQ1), malvidin-3-O-glucoside equivalent 2 (Mv3G EQ2), malvidin-3-O-glucoside equivalent 3 (Mv3G EQ3). Results were expressed as milligrams of gallic acid equivalent/gram of dry weight of skin (mg GAE/g dw).

### 2.3. Determination of Polyphenols by HPLC-DAD Analysis

The analysis of polyphenols was performed using a high-performance liquid chromatography (HPLC) 1100 (Agilent Technologies, Palo Alto, CA, USA) equipped with a diode array detector (DAD). The reversed stationary phase employed was a Zorbax SB C18 5 µm (250 × 4.6 mm i.d., Agilent) with a guard column Zorbax C18 5 µm (35 × 0.5 mm i.d., Agilent). The following gradient system was used with acetonitrile (solvent A) and water/formic acid (90:10, *v/v*) (solvent B): 0–2 min 5% A-95% B; 10 min 13% A-87% B; 20 min 15% A-85% B; 30 min 22% A-78% B; 50 min 22% A-78% B; 55 min 95% A-5% B; 60 min 5% A-95% B, stop time at 65 min. The flow was maintained at 0.7 mL/min and column temperature at 25 °C; the sample injection was 3 µL. Diode array detection was between 250 and 650 nm, and absorbance was recorded at 520, 360, and 280 nm to identify anthocyanins, flavonols, and flavanols, respectively. Quantification was made using as a reference standard malvidin-3-O-glucoside for anthocyanins, quercetin for flavonols, and (+)-catechin for flavanols using ChemStation rev. B.01.03 (Agilent Technologies, Palo Alto, CA, USA). Results were expressed as milligrams/gram of dry weight of skin (mg/g dw).

### 2.4. Cell Lines and GSEs Treatment

Human colon adenocarcinoma derived Caco2 cell line (well-differentiated) (G1–2) (from adenocarcinoma) and SW480 cell line (poorly-differentiated) (G3–4) (from adenocarcinoma grades III–IV) were purchased from the American Type Culture Collection (ATCC) Cell Bank (Manassas, Virginia). Cells were grown in Roswell Park Memorial Institute (RPMI) 1640 medium for Caco2 cells and Dulbecco’s Modified Eagle Medium (DMEM) for SW480 cells, supplemented with 10% fetal bovine serum (FBS), 2 mM glutamine, 100 U/mL penicillin, and 100 μg/mL streptomycin, in monolayer cultures, and incubated at 37 °C in a humidified atmosphere containing 5% CO_2_ in air. The cells were allowed to grow for the established time and then harvested. For the experimental group, Autumn Royal and Egnatia GSEs were added to the medium at increasing concentrations, and the control group received no treatment; the cells were then incubated at 37 °C in a humidified 5% CO_2_ incubator for the times required by the experiments indicated below.

### 2.5. Assessment of Cell Proliferation

Cells have been cultured for 24 and 48 h with increasing concentrations of Autumn Royal or Egnatia GSEs (10, 20, 50, and 80 μg/mL) dissolved in ethanol:water:hydrogen chloride 37% (70:30:1 *v/v/v*), and no treatment for the control group. The proliferative response was measured by colorimetric 3-(4,5 di-methylthiazol-2-yl)-2,5-diphenyltetrazoliumbromide (MTT) assay (Sigma Aldrich; Milan, Italy), a validated test to assay cell proliferation in vitro [31,32,33,34,35].

### 2.6. Extraction of Lipids from Cell Lines for Fatty Acid Analysis by Gas Chromatography

Lipids were extracted from cell pellets treated with increasing concentrations of Autumn Royal or Egnatia GSEs (20, 50, and 80 μg/mL) for 48 h. The Folch extraction method was used with some modifications [36]. Briefly, an aliquot of 100 µL of cell lysate was diluted to 450 µL of an acidified salt solution (H_2_SO_4_ 2 × 10^−4^ M, NaCl 0.1%). Subsequently, 750 µL of methanol and 1.5 mL of chloroform (Sigma-Aldrich, Milan, Italy) were added and the samples were vortexed and centrifuged. The lower layer containing fatty acids was collected and dried by a centrifugal evaporator (Thermo Fisher Scientific, Waltham, MA, USA). The fatty acid methyl esters (FAMEs) were obtained by adding 250 µL of toluene and 750 µL of boron trifluoride in methanol 14% (Sigma-Aldrich, Milan, Italy). After an incubation of 2 h at 80 °C, 1250 µL of NaCl 5% and 250 µL of toluene (Sigma-Aldrich, Milan, Italy) were added to the samples and then centrifuged at 700 g for 10 min at 4 °C. The upper phase obtained containing the FAMEs was picked up and analyzed into gas chromatograph (Thermo Fisher Scientific, Focus GC, Milan, Italy) using ChromQuest 4.1 software (Thermo Fisher Scientific, Focus GC, Milan, Italy), as previously described [37].

### 2.7. RNA Extraction and Quantitative RT-PCR

Total RNA was extracted from Caco2 and SW480 cells after 48 h of treatment with Autumn Royal or Egnatia GSEs (20, 50, and 80 μg/mL) using the Qiagen RNeasy Mini Kit (Qiagen, Hilden, Germany) according to the manufacturer’s instructions. Samples were retro-transcribed and analyzed using real Time-PCR for the evaluation of SCD1 expression on a CFX96 Touch Real-Time PCR Detection System (Bio-Rad Laboratories, Milan, Italy) according to the manufacturer’s instructions. A specific primer for SCD1 (Unique Assay ID: qHsaCED0042705) pre-validated by Prime PCR SYBR Green Assay from Bio-Rad (Milan, Italy) was used, and β-actin gene (Unique Assay ID: qHsaCED0036269) was chosen as a reference gene. ΔΔCt method was used for relative quantification by CFX Manager software 2.1 (Bio-Rad Laboratories, Milan, Italy).

### 2.8. Western Blotting Analysis

Each cellular pellet, control, and cell treated for 48 h with GSEs (20, 50, and 80 µg/mL) was lysed with Ripa buffer supplemented with protease and phosphatase inhibitors (Thermo Scientific, Rockford, IL, USA). Aliquots of 50 µg of total protein extracts were loaded onto 12% precast polyacrylamide gels (Bio-Rad Laboratories, Milan, Italy). Anti-phospho-Rac1/Cdc42 (Ser71), anti-Rac1/Cdc42, anti-Cofilin, anti-SCD1 (M38), and anti-β-actin (Cell Signaling Technology, Beverly, MA, USA) were used as primary antibodies. The proteins immobilized on membranes and incubated with a secondary antibody were analyzed using ChemiDoc XRS apparatus and Image Lab software 5.2.1, (Bio-Rad Laboratories, Milan, Italy).

### 2.9. Migration Assay

Caco2 and SW480 colon cancer cell lines were trypsinized and seeded onto Oris 96 wells plates coated with collagen I (Platypus Technologies, Madison, WI, USA) at a density of 10 × 10^3^ and 50 × 10^3^ cells per well, respectively. Briefly, after 24 h required for cellular adhesion, cells were treated with Autumn Royal and Egnatia GSEs (5, 10, 20, and 50 µg/mL). Cell migration was examined by using an inverted microscope connected to a CCD camera that allowed to take photographs of each well after the stoppers removal (T0) and after 24 h (T1), 48 h (T2), and 72 h (T3). The measurement of the open areas was performed using the Image J software (http://rsb.info.nih.gov/ij/), and the values were then converted in the percentage of migration, with 100% representative of detection zone completely closed, and plotted in the relative graphs realized with GraphPad Prism 5.0 software (Informer Technologies, Inc, Chicago, IL, USA).

### 2.10. Immunofluorescence Staining

Both cell lines, at a density of 10 × 10^3^ cells per well, were treated with Autumn Royal and Egnatia GSEs (10, 25, 50, and 80 µg/mL), for 24 and 48 h. Untreated cells were used as control. After the treatment for each time point and for the control, cells were washed with PBS, and permeabilized for 15 min with 0.5% Triton X-100 in PBS. Then, cells were blocked with 5% normal serum in PBS for 1 h and incubated at 4 °C overnight with the mix of two primary antibodies: mouse monoclonal anti-human Vimentin (diluted 1:250 in Blocking) and rabbit polyclonal anti-human phospho-Paxillin (Tyr118) (diluted 1:200 in Blocking) (Cell Signaling Technology, Beverly, MA, USA). Subsequently, the samples were washed with PBS and incubated for 1 h at room temperature in the dark side with a specific green-fluorescent conjugated secondary IgG Alexa 488 anti-mouse (green) and IgG Alexa 555 anti-rabbit (red) (Thermo Fisher Scientific, Waltham, MA, USA). After washing with PBS, cells were stained using prolong gold antifade reagent containing DAPI (blue). Images were acquired using a 40× objective confocal microscope Eclipse Ti2 by Nikon and the fluorescence were quantified using Image J software (http://rsb.info.nih.gov/ij/), with the number of pixel/area, randomly using five different areas from the single well.

### 2.11. Statistical Analysis

Data on total phenolic content (TPC) and polyphenolic profile (anthocyanins, flavonols, and flavanols) of GSEs were analyzed by paired Student t-test. For all other parameters investigated, the significance of the differences between the control and treated group was evaluated with one-way analysis of variance (ANOVA) and Dunnett’s post-test. Differences were considered as statistically significant with a *p*-value < 0.05. All data are expressed as mean ± Standard Deviation (SD). STATA statistical software, version 15.1 (StataCorp, 4905 Lakeway Drive, College Station, TX 77845, USA) was used.

## 3. Results

Autumn Royal and the new Egnatia red seedless variety, planted and grown in the Apulia region, have a different composition of active substances, including phenols and polyphenols. Table 1 shows total phenolic content (TPC) and polyphenolic profile of extracts obtained from the skins of these two variety of grape. Both TPC and anthocyanins, flavonols and flavanols were significantly higher in Autumn Royal than in Egnatia.

**Figure 1:**
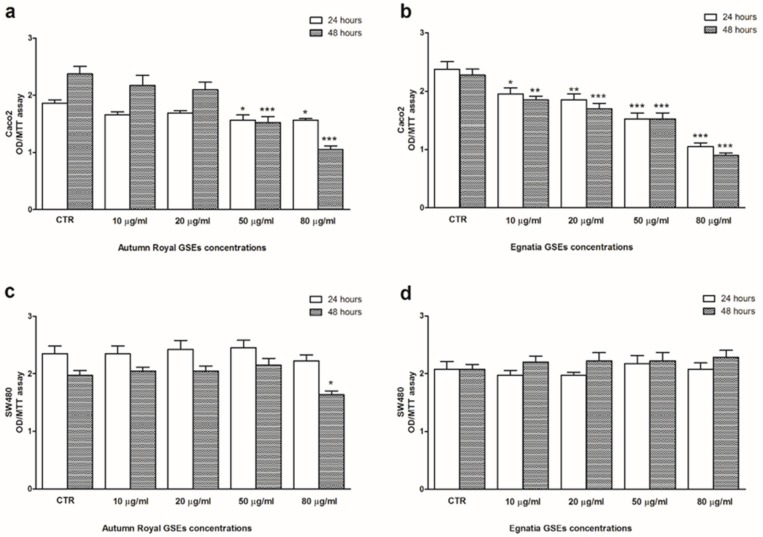
(**a**) Effects on cell proliferation of Caco2 cell line treated with increasing concentrations of Autumn Royal GSEs (10, 20, 50, and 80 µg/mL), for 24 and 48 h of incubation; (**b**) Effects on cell proliferation of Caco2 cell line treated with increasing concentrations of Egnatia GSEs (10, 20, 50, and 80 µg/mL), for 24 and 48 h of incubation; (**c**) Effects on cell proliferation of SW480 cell line treated with increasing concentrations of Autumn Royal GSEs (10, 20, 50, and 80 µg/mL), for 24 and 48 h of incubation; (**d**) Effects on cell proliferation of SW480 cell line treated with increasing concentrations of Egnatia GSEs (10, 20, 50, and 80 µg/mL), for 24 and 48 h of incubation. All data are expressed as the mean ± Standard Deviation (SD) of three consecutive experiments. *p*-value was determined by ANOVA with Dunnett’s post-test. * *p* < 0.05, ** *p* < 0.03 and *** *p* < 0.01 versus control group (CTR).

The effects of Autumn Royal and Egnatia GSEs on cell proliferation of Caco2 (Figure 1a,b) and SW480 (Figure 1c,d) have been assessed by a colorimetric MTT assay. Exposure of the Caco2 cell line to increasing concentration of Autumn Royal GSEs showed an antiproliferative action starting from a concentration of 50 µg/mL, both after 24 and 48 h of treatment (Figure 1a). Egnatia GSEs inhibited cell proliferation already starting from 10 µg/mL, both after 24 and 48 h of treatment (Figure 1b), and this effect was dose-dependent. As regards SW480 cells, the antiproliferative effects were found only by treating cells with high concentrations of Autumn Royal GSEs and exclusively after 48 h of treatment (Figure 1c). Whereas no effect was found in SW480 cells after Egnatia GSEs treatment, both after 24 and 48 h of treatment (Figure 1d).

To investigate the effects of Autumn Royal and Egnatia GSEs on the lipid composition and the fluidity of the cell membranes, we determined the levels of the Stearoyl-CoA desaturase-1 (SCD1) activity after 48 h of treatment of GSEs (Table 2a,b). Compared to the control group in the Caco2 cells, the treatment with increasing concentrations of Autumn Royal and Egnatia GSEs caused an increase in saturated fatty acids (SFAs), starting from the concentration of 50 µg/mL for Autumn Royal and 20 µg/mL for Egnatia (Table 2a). This increase was mainly due to the contribution of the main SFAs, such as palmitic acid (C16:0) and stearic acid (C18:0) for both treatments. On the contrary, a statistically significant reduction of palmitoleic acid (C16:1n7) and oleic acid (C18:1n9) was detected starting at a 20 µg/mL concentration of Autumn Royal and Egnatia GSEs, determining a drastic reduction in monounsaturated fatty acids (MUFAs) compared to the untreated control group (Table 2a). These changes in the lipidomic profile of the Caco2 cell membranes reduced the desaturation indices expressed as palmitoleic acid/palmitic acid and oleic acid/stearic acid ratios, in a dose-dependent manner (Table 2a). The same behavior was observed for the total SCD1 activity, given by the sum of both ratios (Table 2a).

In the SW480 cell line, the treatment with increasing concentrations of GSEs induced minor changes in the composition of membrane fatty acids, probably due to the different basal levels of lipids in the control groups of the two cell lines. A statistically significant reduction was observed only for the oleic acid/stearic acid ratio at the highest concentrations both Autumn Royal and Egnatia (80 µg/mL) (Table 2b). Compared to the untreated control group, an increase in SFAs was found in SW480 cell membranes, in a dose-dependent manner after Autumn Royal treatment, mainly due to the contribution of palmitic acid. A reduction in MUFAs levels was found, starting from the concentration of 50 µg/mL for both treatments, due exclusively to the reduction of oleic acid (Table 2b). As regards the treatment with increasing concentrations of Egnatia, only the higher concentrations (80 µg/mL) induced an increase in SFAs, due exclusively to the contribution of stearic acid (Table 2b). A reduction, although not statistically significant, was found in the total activity levels of SCD1 for SW480 cells treated with increased concentrations of GSEs (Table 2b).

**Figure 2:**
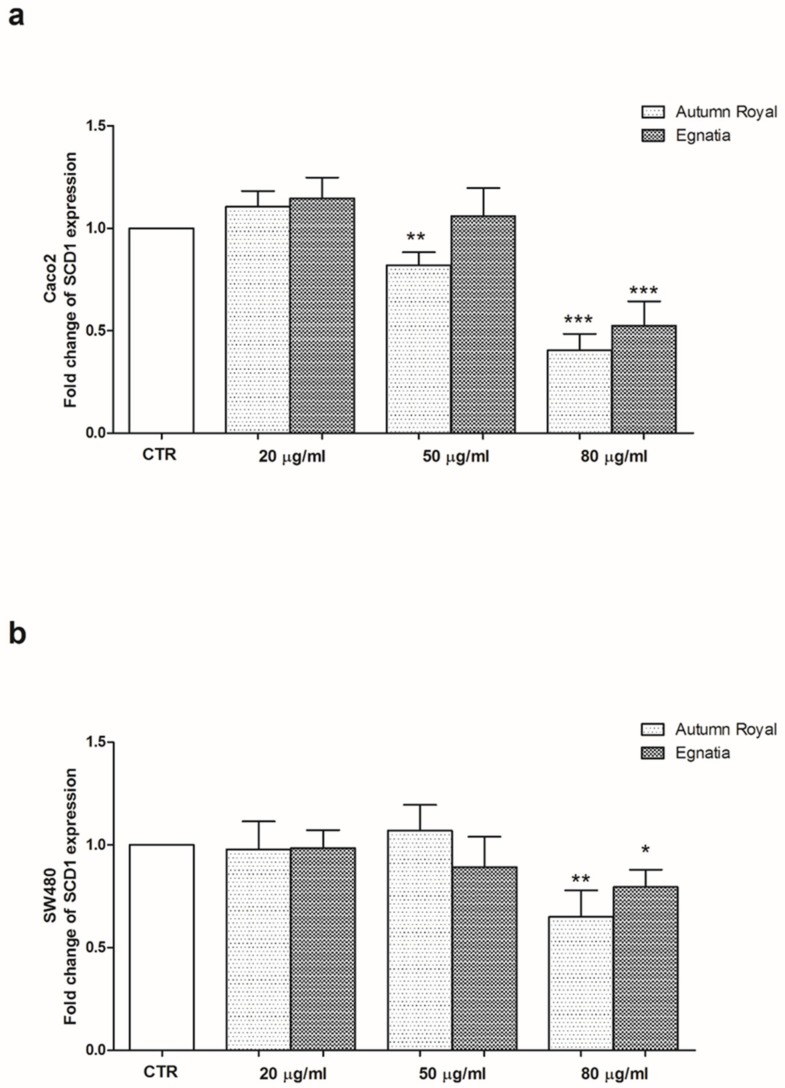
(**a**) SCD1 gene expression levels detected in Caco2 cells treated with increasing concentrations of Autumn Royal and Egnatia GSEs (20, 50, 80 µg/mL) for 48 h of incubation; (**b**) SCD1 gene expression levels detected in SW480 cells treated with increasing concentrations of Autumn Royal and Egnatia GSEs (20, 50, 80 µg/mL) for 48 h of incubation. All data are expressed as mean ± Standard Deviation (SD) of three consecutive experiments. *p*-value was determined by ANOVA with Dunnett’s post-test. * *p* < 0.05, ** *p* < 0.03 and *** *p* < 0.01 versus the control group (CTR).

To better investigate the ability of the two varieties of table grapes, Autumn Royal and Egnatia, to modulate the membrane fluidity, we studied the gene and protein expression of SCD1 and also the expression levels of specific proteins involved in conformational cellular changes. Figure 2 represents the mRNA levels of SCD1 in Caco2 (Figure 2a) and SW480 (Figure 2b) cell lines treated with increasing concentrations of Autumn Royal and Egnatia GSEs (20, 50, 80 µg/mL) for 48 h of incubation. Compared to the control group, a significant down-regulation of SCD1 was detected in Caco2 cells after the treatment of 50 µg/mL of Autumn Royal and 80 µg/mL of Egnatia (Figure 2a). While in SW480 cells, higher concentrations of GSEs (80 µg/mL) are needed to have a reduction of SCD1 expression, demonstrating greater resistance of these poorly differentiated cells to polyphenolic compounds action (Figure 2b).

**Figure 3:**
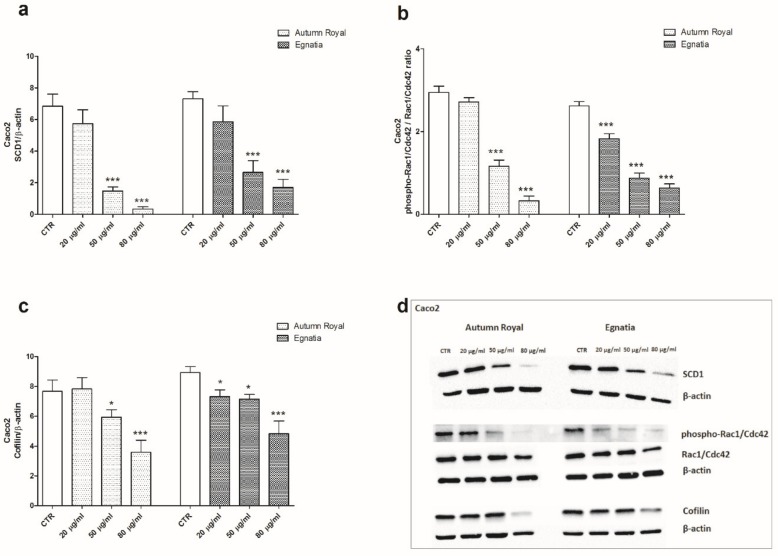
(**a**) SCD1 protein expression levels detected in Caco2 cell line treated with increasing concentration of Autumn Royal and Egnatia GSEs (20, 50, and 80 μg/mL), after 48 h of incubation; (**b**) Phospho-Rac1/Cdc42/Rac1/Cdc42 ratio expression levels detected in Caco2 cell line treated with increasing concentration of Autumn Royal and Egnatia GSEs (20, 50, and 80 μg/mL), after 48 h of incubation; (**c**) Cofilin protein expression levels detected in Caco2 cell line treated with increasing concentration of Autumn Royal and Egnatia GSEs (20, 50, and 80 μg/mL), after 48 h of incubation. All data are expressed as mean ± Standard Deviation (SD) of three different experiments. *p*-value was determined by ANOVA with Dunnett’s post-test. * *p* < 0.05 and *** *p* < 0.01 versus the control group (CTR). (**d**) Representative blots evaluated in Caco2 cells after Autumn Royal and Egnatia treatment.

According to gene expression data, both GSEs were able to reduce the protein expression of SCD1 in Caco2 cell line, starting from concentrations of 50 µg/mL (Figure 3a). To investigate the effects of Autumn Royal and Egnatia GSEs on cell motility, we studied the expression of phospho-Rac1/Cdc42/Rac1/Cdc42 ratio and Cofilin that regulates the actin cytoskeletal reorganization and cellular polarity. Both grape varieties were able to reduce the expression of phospho-Rac1/Cdc42/Rac1/Cdc42 ratio and Cofilin starting from the concentration of 50 µg/mL, compared to the control group (Figure 3b,c, respectively).

**Figure 4:**
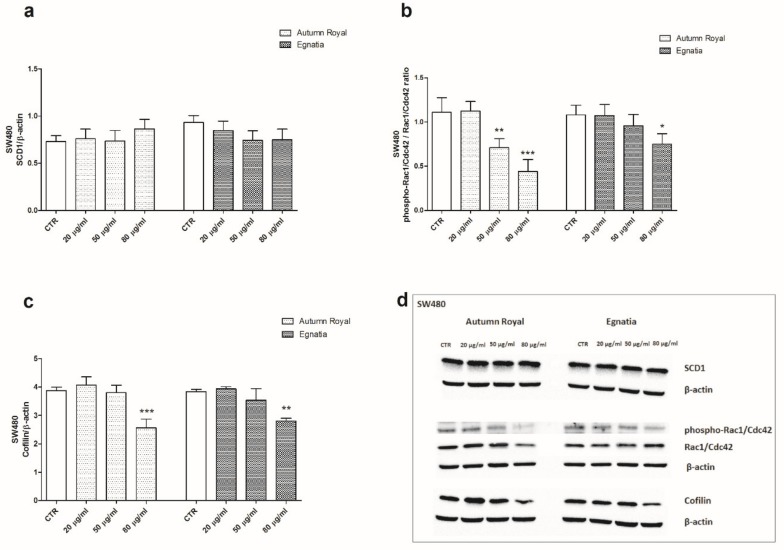
(**a**) SCD1 protein expression levels detected in SW480 cell line treated with increasing concentration of Autumn Royal and Egnatia GSEs (20, 50, and 80 μg/mL), after 48 h of incubation; (**b**) Phospho-Rac1/Cdc42/Rac1/Cdc42 ratio protein expression levels detected in SW480 cell line treated with increasing concentrations of Autumn Royal and Egnatia GSEs (20, 50, and 80 μg/mL), after 48 h of incubation; (**c**) Cofilin protein expression levels detected in SW480 cell line treated with increasing concentrations of Autumn Royal and Egnatia GSEs (20, 50, and 80 μg/mL), after 48 h of incubation. All data are expressed as mean ± Standard Deviation (SD) of three different experiments. *p*-value was determined by ANOVA with Dunnett’s post-test. * *p* < 0.05 and *** *p* < 0.01 versus the control group (CTR). (**d**) Representative blots evaluated in SW480 cells after Autumn Royal and Egnatia.

In SW480 cells, GSEs treatments exerted the same inhibitory effects on phospho-Rac1/Cdc42/Rac1/Cdc42 ratio protein expression starting from the dose of 50 µg/mL (Figure 4b) and on Cofilin protein levels at higher concentrations (80 µg/mL) (Figure 4c). However, no effect was present in SW480 cells on SCD1 protein levels after 48 h of treatments with Autumn Royal and Egnatia GSEs (Figure 4a). This finding suggests that SCD1 modulation could be one of the mechanisms involved in the antitumorigenic action of GSEs.

**Figure 5:**
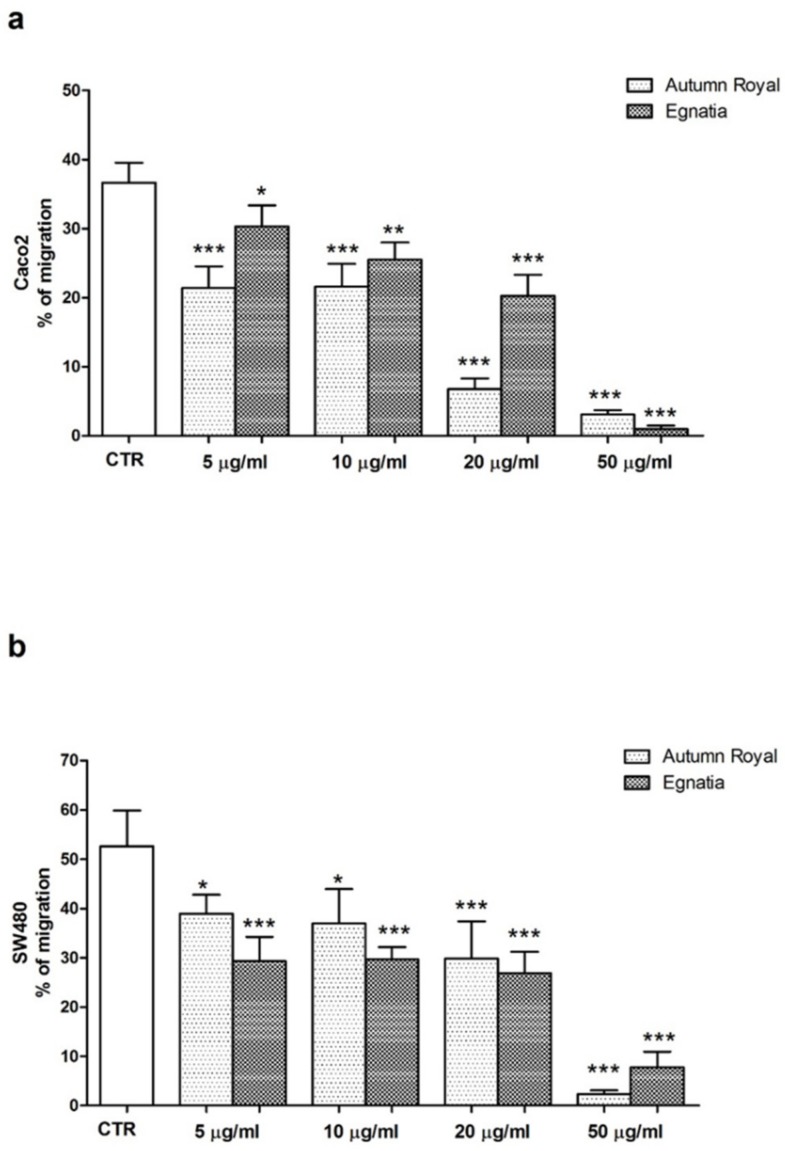
(**a**) Inhibitory effect on migration of Caco2 cells treated with increasing concentrations of Autumn Royal and Egnatia GSEs (5, 10, 20, and 50 μg/mL) for 48 h; (**b**) Inhibitory effect on migration of SW480 cells treated with increasing concentrations of Autumn Royal and Egnatia GSEs (5, 10, 20, and 50 μg/mL) for 48 h. Data represent the percentage of migration expressed as mean ± Standard Deviation (SD) of treated cells respect to control group (CTR) from three independent experiments performed in triplicate. *p*-value was determined by ANOVA with Dunnett’s post-test. * *p* < 0.05, ** *p* < 0.03 and *** *p* < 0.01.

Since both Autumn Royal and Egnatia GSEs influence membrane fluidity and cell motility through the cytoskeleton rearrangement and changes in the membrane fatty acid profile, we investigated whether these extracts may have an additional effect on cell migration. After 48 h of incubation, there was a statistically significant reduction in the percentage of migration starting from the lowest concentration for both treatments in two cell lines (Figure 5a,b). A less evident inhibitory effect was observed after 24 h of treatment, while the same migration behavior was observed after 72 h (data not shown).

**Figure 6:**
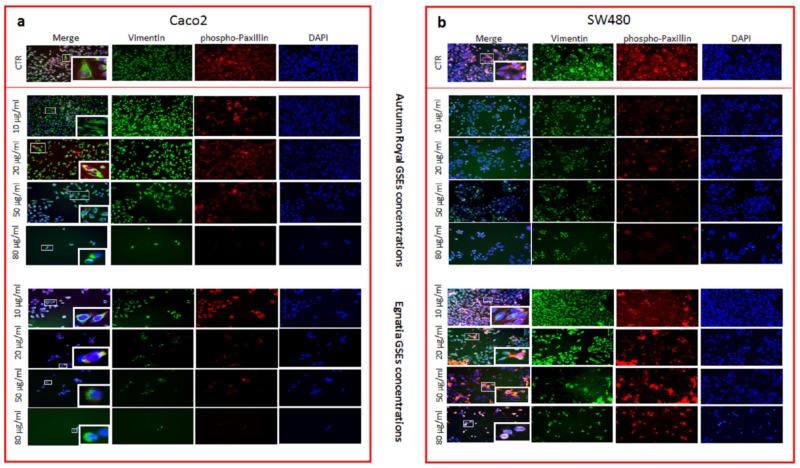
(**a**) Detection of Vimentin and phospho-Paxillin with a 40× objective immunofluorescence confocal microscopy in fixed Caco2 cell lines treated with increasing concentrations of Autumn Royal and Egnatia GSEs (10, 20, 50, and 80 μg/mL) for 48 h; (**b**) Detection of Vimentin and phospho-Paxillin with a 40× objective immunofluorescence confocal microscopy in fixed SW480 cell lines treated with increasing concentrations of Autumn Royal and Egnatia GSEs (10, 20, 50, and 80 μg/mL) for 48 h. Untreated cells were used as control (CTR). Green channel: labeled Vimentin, red channel: labeled phospho-Paxillin, blue channel: labeled nuclei (DAPI), and corresponding overlay (Merge). The images were randomly taken using five different areas from the single well. The enlarged squares indicate the areas of cells to which it refers in the text.

Cellular immunofluorescence images were also performed to evaluate changes in the expression of Vimentin and phospho-Paxillin proteins in both Caco2 and SW480 cell lines (Figure 6a,b, respectively) treated with increasing concentrations of Autumn Royal and Egnatia GSEs (10, 20, 50, 80 µg/mL), at 48 h of treatment. The confocal microscopy images of incubation with GSEs showed a significant reduction in the intensity of green (Vimentin) and red (phosho-Paxillin) fluorescence in the treated cells, compared to the corresponding untreated controls, in a dose-dependent manner. A less noticeable effect was observed at 24 h of treatment (data not shown). Figure 6 shows the morphological cellular changes due to the dose-dependent GSEs treatment. Another evident change in the expression of proteins involved in the organization of the actin cytoskeleton and involved in cell motility was the loss of the fluorescent signal resulting from the reduction of phospho-Paxillin (Figure 6a,b). Furthermore, the fluorescence reduction of cell nuclei (DAPI) confirms the results of the cell proliferation assay (Figure 6a,b).

**Figure 7:**
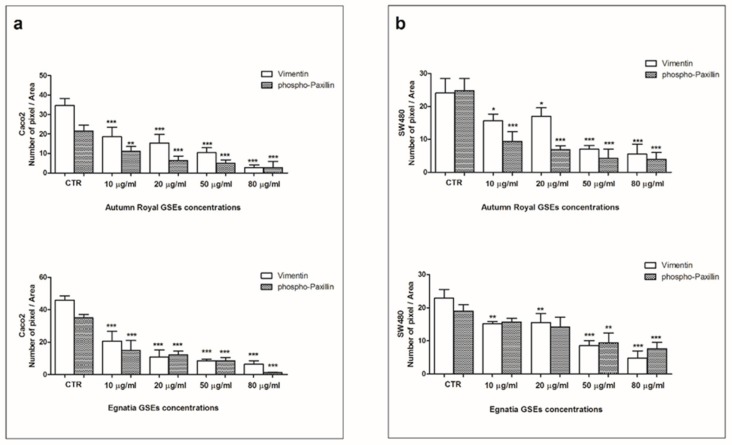
(**a**) Mean intensity index of Vimentin and Phospho-paxillin in fixed Caco2 cell lines treated with increasing concentrations of Autumn Royal and Egnatia GSEs (10, 20, 50, and 80 μg/mL) for 48 h; (**b**) Mean intensity index of Vimentin and Phospho-paxillin in fixed SW480 cell lines treated with increasing concentrations of Autumn Royal and Egnatia GSEs (10, 20, 50, and 80 μg/mL) for 48 h. Immudensitometry calculated by mean intensity index (Number of pixel/Area) of the Vimentin and phospho-Paxillin proteins detected using, randomly, five different areas from the single wall for each single independent experiment performed in triplicate. *p*-value was determined by ANOVA with Dunnett’s post-test. * *p* < 0.05, ** *p* < 0.03 and *** *p* < 0.01 versus control group (CTR).

Compared to the control group, we found a statistically significant reduction in both Vimentin and phospho-Paxillin levels in Caco2 cells, in a dose-dependent manner. The decrease in immunofluorescence levels was statistically significant in both cell lines after 48 h of treatment with increasing concentrations of GSEs. For Caco2 cells, there was a reduction in protein expression levels as early as 10 µg/mL for both Autumn Royal and Egnatia (Figure 7a). While for SW480 cells, Autumn Royal had a greater inhibitory effect on the protein expression of Vimentin and phospho-Paxillin, with respect to the Egnatia variety. With Autumn Royal treatment, there was a reduction of both proteins, while the Egnatia treatment exerted a reduction of Vimentin levels starting from 10 µg/mL and at higher concentrations (50 µg/mL) for phospho-Paxillin, when compared to control group (Figure 7b). A less noticeable effect was observed at 24 h of treatment (data not shown).

## 4. Discussion

GSEs are natural products known for their high anti-tumor properties. Autumn Royal and Egnatia, the new seedless red variety, planted and grown in the Puglia region (Italy), contain high levels of polyphenols, especially anthocyanins, which can reduce cancer cell proliferation and inhibit tumor formation [38]. In this work, we confirmed the antiproliferative activity of table grape polyphenols on two human colon carcinoma cell lines with different degree of differentiation (Caco2 and SW480); in addition, we assessed the ability of these natural compounds to inhibit cell migration by acting on membrane fatty acids composition and the cytoskeletal reorganization and focal adhesion dynamics.

Caco2 cells showed a greater inhibitory action on cell proliferation in a dose-dependent manner after both treatments, with a more pronounced effect for Egnatia compared to Autumn Royal GSEs, both after 24 and 48 h of treatment. In SW480, having a lower grade of differentiation, we observed a lower sensitivity to treatment with both GSEs. However, different behavior of grape extracts on antiproliferative and proapoptotic processes has been previously demonstrated in different human CRC cell lines [39,40,41,42]. In particular, Dinicola et al. [43] have demonstrated that different polyphenols might act additively and/or synergistically to exert total antiproliferative action of the grape extract.

In this work, we have demonstrated the ability of these two table grape varieties to modify the fatty acid profile of cell membranes and the lipids play certainly an important role in the adaptation of cancer cells [7,20]. The treatment with both varieties of GSEs resulted in a reduction in the total levels of SCD1 in the two cell lines studied, given by the sum of the ratios palmitoleic acid/palmitic acid and oleic acid/stearic acid. This inhibitory effect on the desaturation index was pronounced in Caco2 cells, probably due to the different basal levels of fatty acids found between two cell lines.

Several proteomic and lipidomic approaches have shown that in the different types of human cancer cells, there are significant differences concerning specific metabolic enzymes and cellular components, such as membrane fatty acids [44,45]. Although we found very similar levels of total SCD1 in the control groups of the two cell lines, it is important to note that the greatest contribution is given by oleic acid and not by palmitoleic acid. It is known that overexpression of SCD1, and its oleic acid product, is associated with an increase in membrane fluidity in human cancer cells [13,15]. Oleic acid can play an important role in the invasion and metastasis process. In breast cancer cells, this monounsaturated fatty acid promotes an increase in matrix metalloproteinase-9 secretion and invasion through a Protein kinase C, Src, and EGFR-dependent pathway [46]. In this study, we found higher levels of oleic acid in the poorly differentiated cell line SW480 compared to a well-differentiated Caco2 cell line. The increased content of oleic acid, characterizing the membranes of SW480 cells, makes the cell membrane more fluid and more susceptible to homeostasis alterations. The low content of palmitoleic acid, detected in the membrane of untreated control SW480 cells, can probably be justified by the greater transformation of palmitic acid into vaccenic acid catalyzed by elongase 5 (Elovl 5). This evidence is in line with our previous work, in which we demonstrated that high levels of Elovl 5 activity, and consequently high levels of vaccenic acid, were proportionally associated with the severe NAFLD degree [47]. According to our data, other studies have also demonstrated that SCD1 and cis-MUFA are involved in cancer cell progression [48,49]. In vitro and in vivo studies in lung adenocarcinoma have shown that high expression of SCD1 promotes cell invasion and migration, while knockdown of SCD1 significantly reduces carcinogenesis and induces cell apoptosis [50].

In this work, we found that GSEs can reduce the gene and protein expression of SCD1 by decreasing membrane fluidity, such as cell viability and cell migration, in both treated cell lines. A previous in vitro study conducted on HT-29 and SW480 colon cancer cell lines demonstrated the anticancer effects of the polyphenolic compounds present in grape juice extracts from Autumn Royal and Ribier varieties, reducing MMP-2 and MMP-9 gene expression depending on the extract and the cell type [23]. The inhibitory effects of Autumn Royal and Egnatia found on SCD1 expression of Caco2 and SW480 cells were different in relation to the type and of the degree of differentiation of colon cancer cell lines.

The study of fatty acids extracted from cell membranes is considered a valid approach to evaluate the possible effects of natural compounds on cell morphological changes that can influence the different aspects of tumorigenesis [10,19,20,21,22]. In this work, we found that GSEs are able to inhibit cell migration in both human colorectal cancer cells in a dose-dependent manner. However, in SW480 cells, the new table grape variety Egnatia showed a greater inhibitory effect on migration compared to Autumn Royal. This different behavior on cell migration is probably due to the different quality of polyphenolic content between two table grapes. Moreover, a reduction in protein expression levels of phospho-Rac1/Cdc42/Rac1/Cdc42 ratio, Cofilin, Vimentin, and phospho-Paxillin was found in both colon cancer cell lines studied. These proteins are considered cell motility factors because they regulate cell migration through the stimulation and formation of polarized lamellipods, modulation of cytoskeletal organization, focal adhesion turnover, and EMT [51,52,53,54]. There is much evidence that the Rho subfamily proteins, including Rho, Rac, and Cdc42, are involved in actin reorganization and, consequently, in the functioning of cellular processes such as cell migration, cell adhesion, cell polarity, traffic of membranes, and cytokinesis [51,55]. The Rho subfamily regulates the actin cytoskeletal reorganization through Cofilin phosphorylation induced by LIM-kinase family proteins (LIMK1 and LIMK2) [56].

Inside the cell, Vimentin is closely connected to the nucleus, endoplasmic reticulum, and mitochondria, and it is known to play an important role as a support and anchoring of the cytoplasmatic organelles. Moreover, Vimentin plays a crucial role in maintaining the cytoskeletal architecture, and it is able to mediate the microtubule polarity organization, thus inducing tumor cell malignancy [53]. The dissolution of the adherent zonules, together with the expression of mesenchymal proteins, such as the Vimentin, characterize the EMT process [57]. The images obtained with a confocal microscope show a reduction in the fluorescence levels of the Vimentin and phospho-Paxillin proteins, confirming the beneficial role of GSEs on the inhibition of colon cancer progression.

The novelty of the present study is certainly the demonstration that the polyphenolic compounds present in grape extracts can modulate the lipid composition of cell membranes and restore their homeostasis. In addition, here, we confirm the ability of Autumn Royal and the new variety of Egnatia to reduce the motility of human colon cancer cells.

## 5. Conclusions

In conclusion, our data provide novel information concerning the membrane fluidity and cytoskeletal reorganization, also considered key factors in the progression of cancer and the onset of metastasis.

## Figures and Tables

**Table 1:** TPC (total phenolic content) and polyphenolic profile (anthocyanins, flavonols, and flavanols) of Autumn Royal and Egnatia GSEs.

Polyphenolic Compounds	Autumn Royal	Egnatia
TPC (mg GAE/g dw)	63.72 ± 1.10	46.07 ± 2.00 *
Anthocyanins (mg malvidin-3-O-glucoside EQ/g dw)	22.21 ± 2.03	14.19 ± 2.02 *
Flavonols (mg quercetin EQ/g dw)	1.75 ± 0.04	0.27 ± 0.07 *
Flavanols (mg (+)-catechin EQ/g dw)	3.52 ± 0.50	2.48 ± 0.50 *

The TPC was measured by a Folin-Ciocalteu assay and the results were expressed as milligrams of gallic acid equivalent/gram of dry weight of skin (mg GAE/g dw). The analysis of anthocyanins, flavonols, and flavanols were performed by high-performance liquid chromatography (HPLC) with a diode-array detector (DAD), and the results were expressed as milligrams/gram of dry weight of skin (mg/g dw). * *p* < 0.05, paired Student t-test. All values (mean ± Standard Deviation (SD)) were derived from three independent sets of experiments.

**Table 2:** (a) Mean percentage of main saturated and monounsaturated fatty acids in Caco2 membrane cell lines treated with increasing concentrations of Autumn royal and Egnatia GSEs (20, 50, and 80 µg/mL) for 48 h; (b) Mean percentage of main saturated and monounsaturated fatty acids in SW480 membrane cell lines treated with increasing concentrations of Autumn royal and Egnatia GSEs (20, 50, and 80 µg/mL) for 48 h.

Caco2 Fatty Acids (%)		Autumn Royal			Egnatia		
	CTR	20 µg/mL	50 µg/mL	80 µg/mL	20 µg/mL	50 µg/mL	80 µg/mL
Palmitic acid (C16:0)	25.57 ± 1.13	26.22 ± 0.48	26.24 ± 0.46	27.29 ± 0.42 *	27.57 ± 0.40 *	28.91 ± 0.33 *	27.19 ± 0.45 *
Stearic acid (C18:0)	12.98 ± 1.72	13.54 ± 0.54	14.73 ± 0.80 *	16.59 ± 0.63 *	16.08 ± 0.84 *	19.32 ± 0.49 *	19.77 ± 0.69 *
Palmitoleic acid (C16:1n7)	20.67 ± 1.59	19.23 ± 0.31 *	18.67 ± 0.48 *	16.41 ± 0.44 *	15.90 ± 0.67 *	15.08 ± 0.74 *	11.66 ± 0.58 *
Oleic acid (C18:1n9)	17.10 ± 0.68	15.79 ± 0.70 *	14.59 ± 0.56 *	14.29 ± 0.75 *	15.26 ± 0.41 *	14.50 ± 0.61 *	13.65 ± 0.47 *
Saturated fatty acids (SFAs)	42.62 ± 2.52	43.01 ± 0.71	45.07 ± 0.10 *	48.25 ± 0.30 *	46.22 ± 0.78 *	52.31 ± 0.57 *	50.71 ± 0.69 *
Monounsaturated fatty acids (MUFAs)	43.66 ± 2.47	40.92 ± 0.64 *	41.59 ± 0.67 *	36.27 ± 0.58 *	37.38 ± 0.99 *	33.30 ± 0.49 *	30.30 ± 0.68 *
Palmitoleic acid/Palmitic acid ratio (SCD1)	0.81 ± 0.09	0.73 ± 0.02 *	0.71 ± 0.02 *	0.60 ± 0.01 *	0.57 ± 0.02 *	0.52 ± 0.02 *	0.43 ± 0.02 *
Oleic acid/Stearic acid ratio (SCD1)	1.34 ± 0.21	1.16 ± 0.09 *	0.99 ± 0.06 *	0.86 ± 0.07 *	0.95 ± 0.08 *	0.75 ± 0.03 *	0.69 ± 0.04 *
Total SCD1	1.07 ± 0.31	0.95 ± 0.23	0.85 ± 0.15 *	0.73 ± 0.14 *	0.76 ± 0.20 *	0.63 ± 0.12 *	0.56 ± 0.14 *
**SW480 Fatty Acids (%)**		**Autumn Royal**			**Egnatia**		
	**CTR**	**20 µg/mL**	**50 µg/mL**	**80 µg/mL**	**20 µg/mL**	**50 µg/mL**	**80 µg/mL**
Palmitic acid (C16:0)	24.64 ± 0.54	24.15 ± 0.32	26.44 ± 0.36 *	26.82 ± 0.24 *	25.15 ± 0.28	24.49 ± 0.85	24.53 ± 0.54
Stearic acid (C18:0)	13.73 ± 2.48	15.03 ± 2.49	14.97 ± 2.49	18.09 ± 1.58 *	14.32 ± 2.17	16.61 ± 0.48 *	17.29 ± 0.76 *
Palmitoleic acid (C16:1n7)	5.42 ± 1.06	4.63 ± 1.18	4.96 ± 1.18	4.99 ± 1.01	4.74 ± 1.16	4.81 ± 1.72	4.47 ± 1.78
Oleic acid (C18:1n9)	24.13 ± 1.55	23.06 ± 0.53	21.93 ± 0.39 *	20.52 ± 2.01 *	23.89 ± 0.65	23.67 ± 0.67	20.67 ± 1.96 *
Saturated fatty acids (SFAs)	43.48 ± 0.99	44.87 ± 0.66 *	47.14 ± 0.64 *	48.80 ± 0.41 *	42.86 ± 0.30	43.60 ± 0.65	45.42 ± 0.48 *
Monounsaturated fatty acids (MUFAs)	40.05 ± 2.44	38.17 ± 0.34	34.34 ± 0.69 *	32.61 ± 0.58 *	39.15 ± 0.63	37.34 ± 0.98 *	36.37 ± 1.46 *
Palmitoleic acid/Palmitic acid ratio (SCD1)	0.22 ± 0.04	0.19 ± 0.04	0.19 ± 0.04	0.18 ± 0.03	0.18 ± 0.04	0.19 ± 0.07	0.18 ± 0.07
Oleic acid/Stearic acid ratio (SCD1)	1.81 ± 0.39	1.58 ± 0.32	1.50 ± 0.31	1.16 ± 0.06 *	1.71 ± 0.37	1.42 ± 0.07	1.21 ± 0.10 *
Total SCD1	1.02 ± 0.86	0.88 ± 0.75	0.84 ± 0.72	0.66 ± 0.50	0.95 ± 0.83	0.81 ± 0.64	0.68 ± 0.53

Mean value ± Standard Deviation (SD) of three consecutive experiments. *p*-value was determined by ANOVA with Dunnett’s post-test. * *p* < 0.05 versus the control group (CTR).
